# Paraneoplastic Autoimmunity Associated with Testicular Myeloid Sarcoma and Chronic Myelomonocytic Leukemia

**DOI:** 10.1155/2013/656543

**Published:** 2013-10-02

**Authors:** Jeffrey W. Craig, Richard J. Lin

**Affiliations:** ^1^Weill Cornell/Rockefeller/Sloan-Kettering Tri-Institutional MD-PhD Program, 1300 York Avenue, Room C-103, New York, NY 10065, USA; ^2^Department of Medicine, Weill Cornell Medical College, 525 East 68th Street, P.O. Box 130, New York, NY 10065, USA

## Abstract

Myeloid sarcomas are rare extramedullary solid tumors composed of immature myeloid cells. The clinical presentations of these malignant neoplasms are highly variable, ranging from asymptomatic to localized mass effect. Here, we report an unusual case of myeloid sarcoma of the testis found in association with chronic myelomonocytic leukemia where the presenting symptoms were autoimmune pericarditis and migratory arthralgias and myalgias that preceded testicular enlargement by nearly three months. Treatment with both radical orchiectomy and leukemia-directed chemotherapy led to immediate reductions in symptom severity, suggesting that these early symptoms were paraneoplastic in origin. Review of the literature identified the association between hematological malignancies, including chronic myelomonocytic leukemia, and paraneoplastic autoimmune phenomena with features similar to polymyalgia rheumatica and rheumatoid arthritis. Importantly, rheumatologic symptoms related to these disease entities may be easily dismissed as vague or unrelated complaints or treated as purely rheumatologic conditions, thus delaying the formal diagnoses. Clinicians must recognize the common association between possible paraneoplastic rheumatologic symptoms and hematologic malignancies such as chronic myelomonocytic leukemia.

## 1. Introduction

Myeloid sarcomas are tumor masses that consist of myeloid blasts or immature myeloid cells that occur at extramedullary sites throughout the body [1]. While the most commonly affected sites are the skin, lymph nodes, bone, soft tissues, and gastrointestinal tract, these neoplasms may be found at any location and are often accompanied by bone marrow involvement of an underlying hematologic malignancy [2]. Myeloid sarcomas are not associated with a classical presentation but are rather associated with an assortment of clinical findings dependent on tumor size, tumor location, and the direct consequences of underlying hematologic disorders (e.g., infection, bleeding, and organomegaly) [3]. Myeloid sarcomas occur less frequently than many other solid tumors, and the histomorphologic diagnosis is often difficult or delayed in the absence of a previously diagnosed blood-born malignancy [4]. Due to histomorphological similarity to non-Hodgkin's lymphomas, myeloid sarcomas are often initially confused with aggressive large B-cell lymphomas [5]. Obtaining the correct diagnosis is critical, however, as the chemotherapeutic regimens used for treating malignant lymphoproliferative disorders differ substantially from those used for treating acute nonlymphoblastic leukemias. Surprisingly, the clinical behaviors and therapeutic responses of myeloid sarcomas do not appear to be influenced by patient age, gender, anatomic location, individual association with acute myeloid leukemia (AML), myelodysplastic syndrome (MDS), or myeloproliferative disorders (MPD), histomorphological subclassification, or the vast majority of cytogenetic findings [6].

In the largest series of adult-derived myeloid sarcomas reported to date, tumors were found to occur most frequently in the setting of previous or concurrent AML (41%) or as *de novo* neoplasms (27%) and less frequently in association with previous or concurrent MPD (15%) or MDS (15%) [6]. Myeloid sarcomas involving the testis show an even more profound association with AML (68%), while cases associated with MPD, MDS, or chronic myelomonocytic leukemia (CMML) have been documented only rarely [7]. In this report, we describe an unusual case of myeloid sarcoma of the testis, where the initial clinical manifestations included refractory pericarditis and an atypical pattern of migratory arthralgias and myalgias confined to the upper extremities. The diagnosis of myeloid sarcoma in this case was accompanied by the concurrent diagnosis of CMML, and the patient's initial symptoms were attributed to CMML-related autoimmunity owing to the rapid and drastic reduction in symptom severity following treatment [8]. What follows are a succinct description of this unusual case and a discussion of how certain underlying hematologic disorders connected to myeloid sarcomas, namely, MDS and CMML, can further broaden the range of presenting symptoms associated with these neoplasms by virtue of their propensity for eliciting autoimmune rheumatologic phenomena.

## 2. Case Presentation

A 52-year-old man was referred to the emergency department for a 4-week history of painless testicular enlargement by his rheumatologist. Sixteen weeks prior to admission he had been diagnosed with acute pericarditis at an outside hospital, where he received NSAIDs and colchicine. For the ensuing two months, pleuritic chest pain recurred frequently requiring treatment with high dose corticosteroids. Fourteen weeks prior to admission he began experiencing left elbow pain and was treated for tendonitis as an outpatient. Despite treatment, his elbow pain persisted and subsequently spread to the left shoulder and right elbow. By the time of admission, his pain had grown to include the right shoulder and the soft tissues of the upper arms and forearms. His past medical history was otherwise notable only for an asymptomatic normochromic microcytic anemia found incidentally 6 months prior to admission, for which the patient had been prescribed iron supplements. On physical exam his right testis was diffusely enlarged but non-tender and without palpable inguinal lymphadenopathy. His upper extremities were severely tender to deep palpation, most notably over the bony prominences and the soft tissue of the upper arms. The affected areas were not swollen, warm, or erythematous, and active/passive range-of-motion was limited only to pain. An electrocardiogram showed diffuse ST-segment elevations and PR-segment depressions, consistent with unresolved pericarditis. Bedside echocardiogram showed only a small pericardial effusion, stable in size since his pericarditis was first discovered.

His complete blood count (CBC) on admission revealed a normochromic anemia (hemoglobin 8.5 g/dL (range 13.3–17.7), mean corpuscular volume 80.3 fL) with normal platelets and a modest leukocytosis of 14.1 × 10^3^/mL (range 3.4–10.2). Serum lactate dehydrogenase was moderately elevated, however serum creatine kinase and aldolase levels were unremarkable. Rheumatoid factor was trivially elevated, and an antinuclear antibody enzyme immunoassay screen was negative. Erythrocyte sedimentation rate and serum C reactive protein level were significantly elevated, at 95 mm/hr (normal < 20) and 8.46 mg/dL (normal < 1), respectively. Radiographs of the bilateral shoulders and elbows showed no evidence of soft tissue swelling or joint effusion. Doppler ultrasonography of his scrotum revealed an enlarged hypervascular right testicle containing a 5.8 cm underlying vascular hypoechoic mass. The left testicle appeared normal in size. Alpha-fetoprotein and beta human chorionic gonadotropin tumor markers were within normal limits. Computed tomography of his chest, abdomen, and pelvis showed no evidence of metastatic disease but was notable for mild aortocaval lymphadenopathy. Preoperative blood work obtained 5 days following admission was remarkable for an increasing leukocytosis. A manual differential indicated both a relative and absolute monocytosis (2.90 × 10^3^/mL, range 0.2–0.9 × 10^3^/mL), as well as the presence of myelocytes and metamyelocytes. Seven days following admission, the patient underwent an uncomplicated right radical orchiectomy, which was followed by drastic and immediate reductions in his pericardial chest pain and the arthralgic/myalgic pains in his upper extremities. 

**Figure 1:**
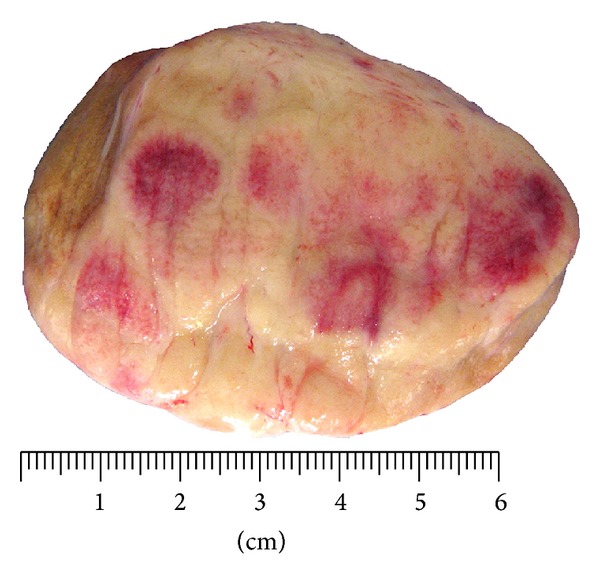
Myeloid sarcoma of the testicle with cut surface appearing as white/tan homogenous tumor with hemorrhage.

**Figure 2:**
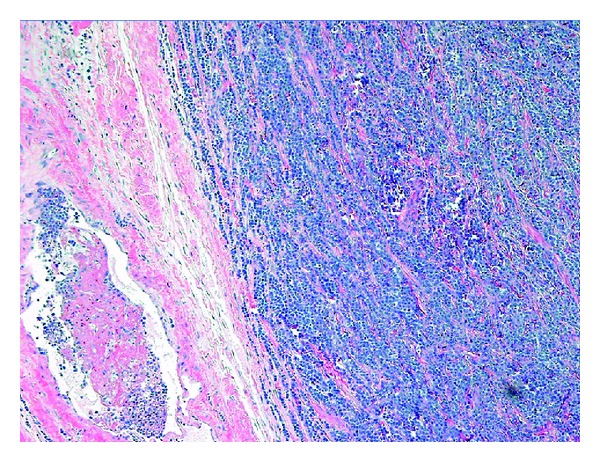
Architectural effacement and replacement of testicle by a diffuse proliferation of immature myeloid cells (H & E stain).

Gross examination of the surgical specimen revealed that the right testis was largely replaced by a firm ill-defined white/tan tumor measuring 6.7 × 5.4 × 3.4 cm. The cut surface appeared largely homogenous with areas of hemorrhage (Figure 1). While the tumor was contained by tunica albuginea, involvement of the rete testis and epididymis was readily apparent. Microscopic examination revealed architectural effacement by a diffuse atypical cell proliferation composed of medium to large sized cells with round to oval nuclei, fine chromatin and conspicuous nucleoli (Figure 2). The atypical cells were positive by immunohistochemical staining for CD45, CD43, CD4, CD56, CD68-KP1, and lysozyme, but were negative for MPO, TdT, CD34, CD117, and all other markers tested. Approximately 90% of the atypical cells were positive by Ki67 immunostaining. The above findings are most consistent with a testicular tumor composed of immature myeloid cells with monocytic differentiation, confirming the diagnosis of myeloid sarcoma [9]. 

**Figure 3:**
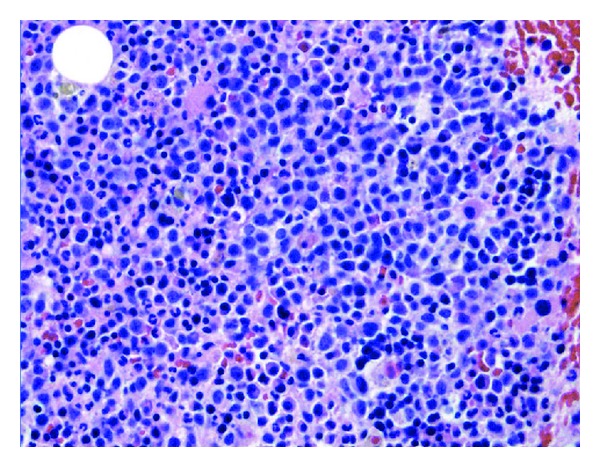
Hypercellular bone marrow with aggregates of large immature mononuclear cells (H & E stain).

The patient's bone marrow was sampled and found to be markedly hypercellular for age, with an increased myeloid: erythroid ratio and full maturation of both lineages. Although there was no increase in CD34-positive blasts, there were dysplastic megakaryocytes and an increased number of CD56/Lysozyme/CD68-KP1-positive large cells, present in aggregates, likely representing immature mononuclear cells (Figure 3). Approximately 25% of this latter cell population also tested positive for non-specific esterase, which further indicated monocytic differentiation. Conventional cytogenetic analysis revealed trisomy 8, a common finding in myeloid sarcomas that may be associated with a poorer prognosis [10]. An interphase FISH assay indicated that 7% of all cells analyzed possessed the trisomy 8 genotype. A bone marrow differential cell count showed 4% blasts (normal 0–3%), 8% monocytes (normal 0–3%), and 17% immature mononuclear cells (normal 0%). Peripheral blood analysis was performed simultaneously, revealing a normochromic anemia (hemoglobin 6.9 g/dL, mean corpuscular volume 83.0 fL) and leukocytosis (17.3 × 10^3^/mL) with a predominance of monocytes (20%) and few blasts (1%). The above findings are consistent with chronic myelomonocytic leukemia (CMML-1; <10% medullary and <5% peripheral blasts), a malignant neoplasm exhibiting both myelodysplastic and myeloproliferative features and characterized by peripheral monocytosis. Even though cases of myeloid sarcoma diagnosed concurrently with MPN, MDS, or CMML are not universally associated with blast crisis and overt transformation to standard AML, myeloid sarcoma occurring in the presence of bone marrow disease is sufficient to establish a clinical diagnosis of AML and to warrant the prompt initiation of AML-targeted therapy [1, 11, 12]. Twenty days into his admission, the patient was given intrathecal methotrexate and started on a 10-day cycle of decitabine induction chemotherapy. Similar to the patient's prior orchiectomy, induction chemotherapy was associated with immediate reductions in his pericardial and upper extremity pains. Unfortunately, his disease was subsequently complicated by an inflammatory vasculopathy with an IgM-aCL-mediated hypercoagulability, as well as multiple infections leading to septic shock. The patient expired approximately 6 months after his original hospital admission to our institution.

## 3. Discussion

The most striking feature of the case presented above is the order in which symptoms first appeared. As solid tumors, myeloid sarcomas often become visible early in their course or cause symptoms related to mass effect. In this case, however, the first symptoms that prompted the patient to seek medical attention were acute pericarditis and arthralgias/myalgias of the upper extremities. These early symptoms began nearly 3 months prior to the first observation of scrotal asymmetry, at a time when there was no clear evidence of underlying hematologic malignancy. Furthermore, these symptoms were rapidly reduced, albeit temporarily, following radical orchiectomy and the initiation of induction chemotherapy, both of which were associated with the immediate bulk elimination of antigenic cancer cells. This finding suggests that the symptoms experienced prior to the first recognition of testicular enlargement represent paraneoplastic manifestations of the concurrently diagnosed CMML. Paraneoplastic autoimmune phenomena are seen in approximately 10% of patients with MDS and CMML and span a range of clinical manifestations including acute systemic vasculitic disease, isolated chronic autoimmune phenomena, classical connective tissue disorders, immune-mediated cytopenias, and serum immunologic abnormalities [8, 13]. For this reason, we strongly suspected that autoimmunity related to the patient's underlying CMML was the likely cause of his earliest symptoms of pleuritic chest pain, arthralgias, and myalgias, as well as the IgM-aCL hypercoagulable state that later complicated his disease course. 

Review of the literature supported the long-established observation that some rheumatologic disorders are associated with or even precede the clinical manifestations of a variety of solid and hematological tumors. Inflammatory myopathies seronegative rheumatoid arthritis lupus-like, scleroderma-like, and polymyalgia rheumatica, and some atypical vasculitides are the most frequently reported paraneoplastic rheumatologic disorders. Generally, their clinical course parallels that of the cancer, and surgical removal of the tumor or its medical treatment usually results in a marked regression of symptoms. Unfortunately, most paraneoplastic rheumatologic disorders are difficult to distinguish from their idiopathic counterparts, and clinicians must rely on atypical features of the clinical presentation to identify the underlying malignancy [14, 15].

In summary, our case of myeloid sarcoma of the testis with underlying CMML highlights the ability of these tumors to initially present with symptoms of paraneoplastic autoimmunity, rather than symptoms directly related to tumor size or primary location. While there are several other reports of testicular myeloid sarcoma arising in the setting of either MDS or CMML [11, 16–18], to the best of our knowledge, this is the first report identifying paraneoplastic autoimmune phenomena as the first and the most striking features of disease.
